# Intratemporal complications of otitis media

**DOI:** 10.5935/1808-8694.20130026

**Published:** 2015-11-02

**Authors:** André Souza de Albuquerque Maranhão, José Santos Cruz de Andrade, Valéria Romero Godofredo, Rafaella Caruso Matos, Norma de Oliveira Penido

**Affiliations:** a MSc in otorhinolaryngology at UNIFESP-EPM (Graduate student in the Department of Otorhinolaryngology at UNIFESP-EPM); b MD (Second-year resident physician in the Department of Otorhinolaryngology at UNIFESP-EPM); c Medical Student (Medical Student at UNIFESP-EPM); d Post doctoral degree in Otorhinolaryngology (Adjunct Professor in the Department of Otorhinolaryngology at UNIFESP-EPM). Federal University of São Paulo - Paulista Medical School (UNIFESP-EPM)

**Keywords:** otitis media, otitis media, suppurative, temporal bone

## Abstract

Otitis media (OM) is considered a potentially severe disease due to the risk of complications.

**Objective:**

To establish the annual incidence of intratemporal complications (ITC) resulting from OM and to prospectively assess patients for epidemiological and clinical factors.

**Method:**

This prospective cohort study included patients admitted during one year at a university hospital diagnosed with intratemporal complications of OM. Patients were analyzed for age, gender, type of intratemporal complication, treatment, and clinical outcome. The overall incidence of complications and the specific incidence rates of each type of complication were determined.

**Results:**

1,816 patients were diagnosed with OM; 592 (33%) had chronic OM; 1224 (67%) had acute OM. Fifteen patients were diagnosed with OM ITC, adding up to an annual incidence of 0.8%. Nineteen diagnoses of ITC were made in 15 patients. Seven (36.8%) patients were diagnosed with labyrinthine fistula, five (26.3%) with mastoiditis, four (21.1%) with peripheral facial palsy, and three (15.8%) with labyrinthitis.

**Conclusion:**

The incidence of intratemporal complications remains significant when compared to the rates seen in developed countries. Chronic cholesteatomatous otitis media is the most frequent etiology of intratemporal complications. Labyrinthine fistula is the most common intratemporal complication.

## INTRODUCTION

Otitis media (OM) is considered a potentially severe disease due to the risk of complications^1^, and should be faced as a dynamic infirmity that ranges from a self-limiting benign condition to a prolonged and often complicated disease. A number of factors impact the form of disease patients develop, such as age, local and systemic immune status, virulence of the causing agent, and previous treatments^2^.

The advent of antibiotics in the last century, more particularly in the 1940s, led to a dramatic decline in the incidence of complications from acute otitis media (AOM) and chronic otitis media (COM), and changed the preferences from surgical treatment to predominantly clinical management of the disease^3^, ^4^.

A meeting of the World Health Organization (WHO) on health policies for chronic middle ear infections held in 1998 defined OM as an important and permanent public health issue, specifically in less favored communities in developing and developed countries. The WHO also verified the scarcity of population data on the matter, and called countries to compile epidemiological information to determine the impact of OM and set the priorities for prevention and treatment of the condition^5^. Brazilian epidemiological data publications are scarce, and adapting foreign data to the country's reality is imprecise and less than rigorously scientific.

This paper aims to establish the annual incidence of intratemporal complications associated with otitis media and to prospectively assess the involved patients by analyzing epidemiological and clinical factors.

## METHOD

This longitudinal prospective cohort study included patients diagnosed with acute and chronic otitis media and subjects with intratemporal complications arising from OM seen at an emergency care unit, an outpatient ward, and a university hospital from February of 2010 to January of 2011. Patients with complications were followed up while they were hospitalized and seen in an outpatient setting. Patients with intratemporal complications were analyzed for the following factors: age, gender, type of intratemporal complication, treatment, imaging findings, degree of peripheral facial palsy (House-Brackmann score^6^), type and degree of hearing loss (during the course of infection and after treatment), and clinical outcome. Overall and specific complication incidence rates were calculated. Intratemporal complications were defined as follows: labyrinthine fistula - erosion of the endochondral bone overlying the labyrinth (seen on CT scans or surgery) without perilymph leakage; mastoiditis - erythema, edema/collapsing edema (including subperiosteal abscess); peripheral facial palsy - as per the House-Brackmann score definition; labyrinthitis - worsened hearing thresholds (reported by patient) verified by audiometry (drop in bone conduction) and followed by vestibular signs and symptoms. Patients with uncomplicated otitis had the following data analyzed: age, gender, otological diagnosis (AOM or COM). This study was approved by the institution's Research Ethics Committee and given permit 0081/10.

## RESULTS

In the twelve months covered by the study, 1.816 patients were diagnosed with OM. Eight hundred and seventy-three (48%) were males and 943 (52%) females. The subjects had a mean age of 31 years (0-99 years). COM was diagnosed in 592 (33%) patients, while AOM was seen in 1224 (67%). A ratio of 2:1 was observed between AOM and COM.

Fifteen of the 1816 patients included in the study had intratemporal complications associated with otitis media, yielding an annual incidence rate of 0.8%. Nine (60%) of the 15 subjects were females and six (40%) were males. Chronic cholesteatomatous otitis media (CCOM) was diagnosed in 11 (74%) patients, AOM in three (20%), and non-cholesteatomatous chronic otitis media (NCCOM) in one (6%) subject. Mean age was 52 years (26-78 years).

Two of the fifteen individuals with intratemporal complications had two concurrent complications and one subject had three concurrent complications, adding up to 19 diagnosed cases of ITC. The incidence of each complication is listed in Table 1. One patient had an associated intracranial complication (sigmoid sinus thrombosis). All patients were given intravenous antibiotics and steroids while hospitalized.

**Table 1:** Intratemporal complications of 15 patients in a total of 19 diagnosed complications (two patients had two concurrent complications and one had three concurrent complications).

Intratemporal complication	n	%
Labyrinthine fistula	7	36.8%
Mastoiditis	5	26.3%
Peripheral facial palsy	4	21.1%
Labyrinthitis	3	15.8%
Total	19	100.0%

The distribution of the otological diagnoses of the 19 complications can be seen on Table 2.

**Table 2:** Table 2. Distribution of complication diagnosis (n = 19).

	Labyrinthine fistula (n = 7)		Mastoiditis (n = 5)		Peripheral facial palsy (n = 4)		Labyrinthitis (n = 3)		Total (n = 19)	
	n	%	n	%	n	%	n	%	n	%
AOM	-	-	2	40.0%	2	50.0%	2	66.7%	6	31.6%
NCCOM	1	14.3%	-	-	-	-	1	33.3%	2	10.5%
CCOM	6	85.7%	3	60.0%	2	50.0%	-	-	11	57.9%
Total	7	100.0%	5	100.0%	4	100.0%	3	100.0%	19	100.0%

AOM: Acute otitis media; NCCOM: Non-cholesteatomatous chronic otitis media; CCOM: Chronic cholesteatomatous otitis media.

Canal wall down mastoidectomy was performed in 10 (65%) patients; seven (47%) had undergone mastoidectomy previously and four (57%) had been submitted to more than one mastoidectomy procedure. These patients had been diagnosed with CCOM. The approaches are shown in Table 3. Comorbidities were present in nine (60%) patients. The more prevalent were systemic hypertension (SH) and diabetes mellitus (DM).

**Table 3:** Treatments offered to patients with complications (antibiotics and steroids were prescribed to all patients).

Treatment	n	%
Canal wall down mastoidectomy	10	66.7%
Closed mastoidectomy	2	13.3%
Retroauricular drainage	1	6.7%
Ventilation tube	1	6.7%
Conservative	1	6.7%
Total	15	100.0%

The signs and symptoms occurred prior to complication for each diagnosis are listed on Table 4.

**Table 4:** Incidence of signs and symptoms preceding complications (%), for each diagnosis.

	AOM (n = 3)		NCCOM (n = 1)		CCOM (n = 11)		Total (n = 15)	
	n	%	n	%	n	%	n	%
Otorrhea	3	100.0%	-	-	11	100.0%	14	93.3%
Hypacusis	3	100.0%	1	25.0%	9	81.8%	13	86.7%
Tinnitus	2	66.7%	1	25.0%	8	72.7%	11	73.3%
Otalgia	3	100.0%	1	25.0%	4	36.4%	8	53.3%
Vertigo	1	33.3%	1	25.0%	5	45.5%	7	46.7%
Nausea/vomiting	1	33.3%	-	0.0%	2	18.2%	3	20.0%
Nystagmus	1	33.3%	-	0.0%	2	18.2%	3	20.0%

AOM: Acute otitis media; NCCOM: Non-cholesteatomatous chronic otitis media; CCOM: Chronic cholesteatomatous otitis media.

### Labyrinthine fistula

Seven patients were diagnosed with labyrinthine fistula, four (57%) males and three (43%) females. This complication had an annual incidence of 0.38%. Patient mean age was 59 years, with ages ranging from 44 to 78. Six (86%) patients were diagnosed with CCOM and one (14%) subject had NCCOM. Six (86%) patients had the disease for over five years. Table 5 contains a summary of the prevalences of signs and symptoms. Hypacusis and tinnitus were seen in all patients.

**Table 5:** Incidence of signs and symptoms in patients with labyrinthine fistula.

Sign/symptom	n	%
Hypacusis	7	100.0%
Tinnitus	7	100.0%
Otorrhea	6	85.7%
Vertigo	5	71.4%
Nystagmus	2	28.6%
Nausea/vomiting	2	28.6%

**Figure 1:**
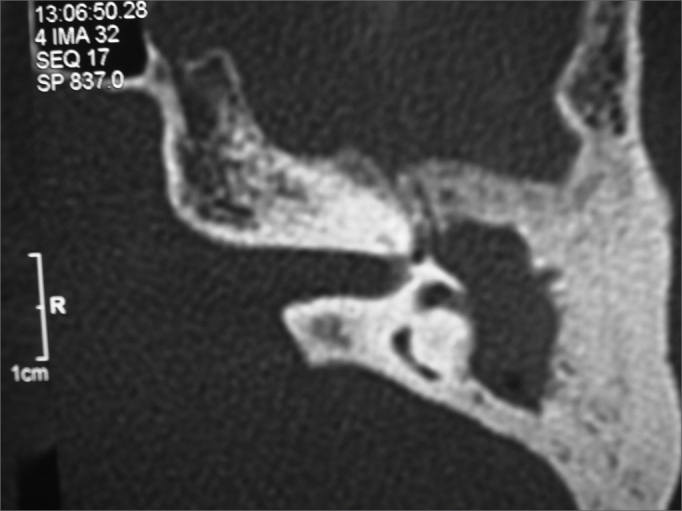
Left-side temporal bone CT scan, axial cross-section, showing blurred antrum and erosion of the lateral semicircular canal.

The nystagmus seen in two (29%) patients was triggered by intense sounds or pressure changes in the ear canal. Observed clinical manifestations, confirmation temporal bone CT scans, and intraoperative findings suggested the diagnosis of labyrinthine fistula. CT scans showed lateral semicircular canal (SCC) erosion in all patients in association with imaging findings consistent with CCOM (Figure 1).

Surgery was the treatment of choice for all patients, and five (84%) subjects underwent canal wall down mastoidectomy. One patient had associated labyrinthitis. Patients were hospitalized for a mean of six days (3-21 days).

A female patient aged 59 years without history of chronic effusion complained of hypacusis and intense left ear tinnitus associated with vertigo lasting for four weeks. Otoscopic examination revealed her eardrum was retracted. She was being treated for diabetes mellitus and had undergone chemotherapy for lymphoma for 11 years. Temporal bone CT scans revealed a tumor with rounded edges occupying the antrum with lateral semicircular canal erosion. The patient was referred to surgery, as she was suspected to have CCOM. During surgery a tumor of fibroelastic consistency was seen in the antrum, along with adjacent mucosal inflammation. A biopsy was done and the pathologist's report read chronic inflammatory process. Immunohistochemistry was performed on the surgical specimen and the patient was diagnosed with an inflammatory myofibroblastic tumor. The patient was started on steroids and her symptoms and hearing thresholds improved significantly.

Preoperative audiometry revealed all subjects had mixed hearing loss, which was moderate in 43% (3/7) of the cases, severe in 43% (3/7), and mild in 14% (1/7). Postoperative audiometry showed persisting mixed hearing loss. Two (27%) patients improved their tone thresholds by a mean of 20 dB at 500, 1000, and 2000 Hz, while one (14%) subject had his thresholds worsened by 15 dB at the frequencies mentioned above.

### Mastoiditis

Five patients were diagnosed with mastoiditis, two males and three females. They had a mean age of 44 years (26-66 years). The annual incidence of mastoiditis was 0.27%. Three (60%) patients were diagnosed with CCOM and two (40%) with AOM. All patients with CCOM had diagnosed disease for at least five years. The time between the onset of symptoms and the patients looking for medical care ranged between five and 33 days (mean = 25).

The encountered signs and symptoms and their incidence rates are shown on Table 6. Otalgia and otorrhea were seen in the five (100%) patients and tinnitus in three (60%).

**Table 6:** Incidence of signs and symptoms in patients with mastoiditis.

Sign/symptom	n	%
Otorrhea	5	100.0%
Otalgia	5	100.0%
Tinnitus	3	60.0%

Canal wall down mastoidectomy was offered to two (40%) patients, closed mastoidectomy to one (20%), retroauricular drainage to one (20%), and conservative treatment (antibiotics) to one (20%) subject.

One patient (20%) had associated intratemporal complications after the start of treatment with antibiotics for mastoiditis and developed peripheral facial palsy (PFP) and labyrinthitis. He underwent tympanocentesis and had a ventilation tube inserted. During hospitalization the patient improved from the symptoms and was referred to a closed mastoidectomy. The patient was hospitalized for 32 days and after intense investigation was diagnosed with Wegener's granulomatosis (WG). The subject was started on cyclophosphamide and steroids, and improved completely from PFP and otorrhea.

Another patient had associated intracranial complication. The subject had been treated for AOM for 30 days when she came to our service complaining of ear pain and otorrhea. Contrast-enhanced CT scans of the temporal bones revealed opacified mastoid air cells and sigmoid sinus thrombosis. Magnetic resonance angiography scans confirmed the diagnosis. The patient was seen by the neurology and ophthalmology teams (to rule out otitic hydrocephalus), but she did not have signs of intracranial hypertension and her ocular fundus exam was normal. She was given broad spectrum antibiotics (ceftriaxone and clindamycin) associated with steroids, and evolved satisfactorily. The patient was in hospital for seven days and was discharged on a 21-day course of cefuroxime axetil.

Two (40%) patients had systemic hypertension. The patients were hospitalized for a mean of 12 days (4-32 days).

### Peripheral facial palsy

Three (75%) of the four individuals diagnosed with PFP were females. Subject mean age was 46 years, ranging from 26 to 64. The annual incidence of PFP was 0.22%. Two (50%) patients were diagnosed with CCOM and two (50%) with AOM. Patients with AOM endured symptoms for a mean of 30 days before they came to our service. Subjects with CCOM waited for 10 days before seeking help.

One patient with mastoiditis developed grade IV PFP (House-Brackmann) and labyrinthitis. This patient was diagnosed with Wegener's granulomatosis based on a positive c-ANCA test, but evolved satisfactorily with complete remission from PFP and proper management of the infection after specific treatment was initiated (as previously described). Another patient also had PFP and labyrinthitis. She had been started on antibiotics 30 days before for AOM and evolved well for two weeks, when hypacusis, tinnitus, and vertigo associated with PFP set in. The patient was on insulin for diabetes (right-eye amaurosis due to diabetic retinopathy) and was being treated for hypothyroidism. She had grade IV PFP (House-Brackmann) and audiometry indicated severe mixed hearing loss. The patient was hospitalized, her antibiotics changed, steroid therapy was strictly managed and glucose levels controlled, and a myringotomy was performed with ventilation tube insertion. The patient was hospitalized for 10 days. When the patient was stabilized, she was discharged on decreasing dosages of steroids. Three months into follow-up the patient presented significant regression from PFP (grade II), reported improvement from tinnitus and vertigo, and her audiometry revealed moderate mixed hearing loss.

Three (75%) patients had grade IV and one (25%) had grade III PFP (House-Brackmann). Two (50%) subjects had other associated complications, a case of mastoiditis and another of labyrinthitis. Mean hospital stay length was 13 days, and ranged from two to 32 days. Two individuals had canal wall down mastoidectomy procedures, one had a closed mastoidectomy, and one a myringotomy with ventilation tube insertion. Three patients evolved favorably from PFP, as seen on Table 7.

**Table 7:** Data from patients with PFP.

Age	Comorbidity	Diagnosis	PFP grade (before)	Treatment	PFP grade (after)
26	None	CCOM	IV	Canal wall down mastoidectomy	III
31	*	AOM	IV	Closed mastoidectomy	I
63	DM; Hypothyroidism	AOM	IV	Myringotomy + VT	II
64	DM	CCOM	III	Canal wall down mastoidectomy	III

^*^ Patient diagnosed with Wegener's granulomatosis as a baseline condition; AOM: Acute otitis media; CCOM: Chronic cholesteatomatous otitis media; PFP: Peripheral facial palsy; DM: Diabetes mellitus; VT: Ventilation tube.

### Labyrinthitis

There were no cases of isolated labyrinthitis. Three patients had labyrinthitis, adding up to an annual incidence of 0.1%. All had associated complications, as shown on Table 8. Hypacusis, tinnitus and vertigo were universally present and nystagmus was observed in two cases.

**Table 8:** Data from patients with labyrinthitis.

Age	Comorbidity	Diagnosis	Audiometry (before)	Associated complication	Treatment	Audiometry (after)
					Closed	Moderate mixed
31	*	AOM	Severe mixed hearing loss	Mastoiditis; PFP	mastoidectomy	hearing loss
59	DM#	NCCOM	Severe mixed hearing loss	Labyrinthine fistula	Closed mastoidectomy	Moderate mixed hearing loss
63	DM; hypothyroidism	AOM	Moderate mixed hearing loss	PFP	Myringotomy and VT	Moderate mixed hearing loss

^*^ Patient diagnosed with Wegener's granulomatosis as a baseline condition;

^#^ Patient had history of lymphoma; AOM: Acute otitis media; NCCOM: Non-cholesteatomatous chronic otitis media; PFP: Peripheral facial palsy; DM: Diabetes mellitus; VT: Ventilation tube.

The three patients mentioned above took steroids for four weeks in decreasing dosages. Two months after treatment the patients reported complete improvement from vertigo and tinnitus and partial improvement from hypacusis. Audiometry tests showed improvements of 10 to 20 dB at 500-1000 and 2000 Hz and residual mixed hearing loss.

## DISCUSSION

This study found an annual incidence rate of intratemporal complications from OM of 0.8%, as also seen in other studies carried out in developing countries such as Thailand^7^, Turkey^8^, and Taiwan^9^, in which rates of 0.45%, 1.35% and 3% were respectively reported. The apparently insignificant reported rates gain weight as they are compared to the incidences seen in developed countries. For example, a study done in Finland^10^ reported an incidence of complications of 0.004%. We believe that troubled access to the public health care system, precarious service, and lack of education promote the continuity of this situation, as discussed by other authors^11^, ^12^.

COM was the most prevalent diagnosis, CCOM in particular, as it accounted for 74% of the complications. Although our sample featured a small number of patients (n = 15), this pattern was also seen in studies with larger cohorts^7^, ^8^.

Three (20%) individuals had two or more intratemporal complications, and one patient had an associated intracranial complication (sigmoid sinus thrombosis). Concurrent complications have been abundantly reported in the literature^7^, ^8^, ^11^, ^13^, stressing the need to meticulously investigate patients with complications for associated complications.

The signs and symptoms presented by patients with complications (Table 4) were divided between subjects with complications stemmed from AOM and individuals with complications arising from COM (NCCOM and CCOM), as they are significantly different from each other. When looking at patients with AOM, we found that otalgia was present on 100% of the patients. By their turn, only 33% of the patients with COM had otalgia. The signs and symptoms of patients with complications from COM are typically nonspecific^9^, such as hypacusis and tinnitus, seen in 84% and 75% of these subjects respectively. They are also older and have associated comorbidities. Early diagnosis and complication prevention require careful patient examination.

### Labyrinthine fistula

Labyrinthine fistula was the most frequently seen intratemporal complication (37%), with an annual incidence of 0.38%. It is defined as erosion of the endochondral bone overlying the labyrinth without perilymph leakage (unlike perilymphatic fistulae), and still is one of the most common complications in CCOM^8^. The incidence of this complication is thought to be underestimated. Studies on the complications of otitis media do not include all cases of labyrinthine fistula, as it does not produce significant symptoms and is detected only during surgery^12^.

Many authors do not include labyrinthine fistula in the differential diagnosis of intratemporal complications, as it usually does not produce exuberant clinical manifestations and its symptoms are nonspecific. Considering this study's most commonly seen signs and symptoms minus labyrinthine fistulae, otalgia, a symptom that certainly draws more attention from physicians as a sign of possible complications, would move up from fourth to second position, with an incidence of 75%, behind only otorrhea, seen in all cases. However, we believe labyrinthine fistula should be included, as it is a condition in which the labyrinth is exposed by the disease, consequently leaving vestibular function and hearing more vulnerable.

Although no intracranial complications from labyrinthine fistulae were seen in our study, they may occur from the communication between the labyrinth and the subarachnoid space, with meningitis being the most frequently reported complication^7^, ^8^, ^9^. Meningitis may prevent the occurrence of other complications, intracranial ones in particular, when treated in time. The data gathered in this study serves as warning to the scientific community and ENTs in particular of the importance of identifying and treating labyrinthine fistulae before they evolve to perilymphatic fistulae.

Eighty-six percent (6/7) of the patients had CCOM, and in all cases the disease had been diagnosed for over five years and for over ten years in 72% (5/7) of the subjects, characterizing a pattern of slow insidious development. It is hard to define when the cholesteatoma appeared, but using the date of disease manifestation, usually in the form of ear effusion, reported by the patient as a reference, one may infer that the disease had been around for even longer. The resorption of the ear capsule is believed to be an insidious process (as previously mentioned), driven by inflammatory mediators activated on the matrix of the cholesteatoma or by pressure applied by the cholesteatoma^14^.

The erosion of the bone layer protecting the labyrinth allows pressure changes to be transmitted to the underlying endosteum, perilymph, and the adjacent endolymphatic compartment, thus evoking vestibular and even auditory symptoms. Only 29% (2/7) of the patients with labyrinthine fistula had nystagmus induced by pressure changes in the ear canal (Hennebert sign) and intense noise (Tullio phenomenon). The literature reports slightly higher incidence rates, indicating that 32% to 50% of the patients tested positive for fistula will have it found during exploratory surgery^14^. Although the presence of sensorineural hearing loss, vertigo, and positive test for fistula suggest labyrinthine fistula, the absence of these findings does not necessarily mean the ear capsule is intact. Therefore, it is prudent to consider fistula in the examination of all patients to undergo surgery.

In our study, the lateral semicircular canal was involved in 100% of the individuals. Given its location close to the antrum, it is the most commonly involved portion of the labyrinth and, as reported by other authors, it accounts for approximately 90% of the cases^12^, ^14^.

Eighty-six percent (6/7) of the patients had CCOM and underwent canal wall down mastoidectomy. Closed mastoidectomy was offered to one individual with NCCOM. Four patients (58%) had previously undergone mastoidectomy. Labyrinthine erosion was observed in all cases. The cholesteatoma matrix was removed in two cases, when a safe cleavage plane was found.

Surgical approaches have been broadly discussed in the literature. Some authors believe the best procedure is canal wall down mastoidectomy, removing the cholesteatoma and keeping the fistula covered by the matrix and moving it out into the cavity^15^, ^16^. These authors believe that the complete removal of the matrix increases the risk of sensorineural hearing loss. The advocates of the closed approach claim it reduces the potential risk of bone erosion progression and infectious complications such as labyrinthitis. However, revision surgery is needed^17^, ^18^. Canal wall down mastoidectomy (removing the cholesteatoma matrix whenever possible) was performed on all patients in our study because of the socio-economic characteristics of the population seen in our practice, the significant extension of the cholesteatomas at the time of surgery, and the difficulty scheduling revision surgery^19^.

### Mastoiditis

Described classically as the most frequent complication of otitis media, mastoiditis ranked second in our study, accounting for 28% of the diagnosed complications. This happened because labyrinthine fistulae were included in the differential diagnosis of intratemporal complications.

The annual incidence of mastoiditis seen in this study was 0.27%. Despite its declining incidence rates, mastoiditis is still very much a part of our reality. In developing countries, mastoiditis and other complications are still the most common cause of death for COM^20^.

The most frequently seen signs and symptoms were otalgia and otorrhea, observed in 100% of the patients. Mastoiditis needs to be correctly defined so it can be properly treated. In general terms, mastoiditis has been defined as mucosal thickening or mastoid cavity effusion. It occurs frequently in cases of AOM and COM and may be seen routinely in temporal bone CT scans. It is not a very significant clinical entity. However, clinical mastoiditis - the entity described in this study - accompanied by erythema, edema, and at times by collapsing edema characterizing subperiosteal abscess, is a different story^12^. Meticulous investigation is required in these cases in order to determine the most adequate course of treatment. Although diagnosis is imminently clinical, temporal bone CT scans, preferably contrast-enhanced, play an important role in planning therapy and ruling out other possible complications.

Although each type of complication arising from OM has a specific therapy, we believe certain treatment principles can be generalized. First, one must define whether the patient has AOM or COM. Practically all cases of complication from AOM evolve well with proper antibiotics and myringotomy with or without ventilation tube insertion. When complications stem from COM, broad spectrum antibiotics effective against anaerobic and aerobic organisms are required, along with some form of mastoidectomy in most cases^12^. Canal wall down mastoidectomy was offered to all three patients with mastoiditis diagnosed with CCOM. One of these patients had an aerated cheekbone and developed an abscess in the zygomatic process. The incision was planned to include the area of the abscess, as recommended in the literature^14^. After draining the abscess, the mastoidectomy was performed and the cholesteatoma removed the usual way.

The two cases of AOM-associated mastoiditis had complications, two intratemporal (PFP and labyrinthitis) and one intracranial (sigmoid sinus thrombosis). Both had otalgia and otorrhea and had taken oral antibiotics, but attained only partial resolution of symptoms. They sought our service 30 days after the onset of clinical manifestations with worsening symptoms. These cases show how the timing of the infection is strongly suggestive of mastoiditis. Marked otalgia or purulent otorrhea persist for two or more weeks, or symptoms recur after a period of apparent clinical improvement^4^, ^12^.

The patient with sigmoid sinus thrombosis from AOM improved clinically with intravenous antibiotics administered in the hospital and was discharged on oral antibiotics for another three weeks. This case should serve as a warning to ENTs for the possibility of sigmoid sinus thrombosis without clinical manifestations and the benign progression of this condition. Because of these characteristics, the treatment of sigmoid sinus thrombosis has been discussed to form a trend towards more conservative therapies^13^, ^21^, i.e., not approaching the sigmoid sinus directly and treating the baseline infectious disease effectively.

### Peripheral facial palsy

Two of the patients with PFP were diagnosed with AOM and two with CCOM. In this study, the annual incidence of PFP as an OM complication was 0.22%. Although seemingly insignificant, it is similar to the incidence rates seen in the pre-antibiotics era of 0.5-0.7%^22^, ^23^. Current incidence in developing countries revolves around 0.005%, according to a study carried out in Denmark^24^.

Peripheral facial palsy (PFP) may result from AOM and COM. Nerve insult usually occurs from congenital dehiscence of the facial nerve canal or by bone erosion caused by granulation tissue or cholesteatoma, which allow inflammatory mediators to compromise nerve function through a process known as suppurative neuropraxia^12^, ^14^. Different theories have been proposed for PFP associated with AOM. Because of its shorter course of progression, it is believed that bone erosion and compressive phenomena are less likely, and some authors believe that nerve injury occurs by the congestion of small vessels triggered by inflammatory processes, resulting in neural ischemia^25^.

The two patients with AOM had associated intratemporal complications, namely labyrinthitis and mastoiditis. Hydén et al. ^26^ described seven cases of concurrent PFP and labyrinthitis in AOM patients, and postulated that the invasion of toxins or infectious agents, probably through the round window membrane, was accompanied by inflammatory nerve injury. Other authors have described strong correlations between AOM and development of PFP and labyrinthitis^10^.

The patient with Wegener's granulomatosis (WG) spent 32 days in hospital before being diagnosed. Various authors have written about the importance of ruling out WG, a rare condition that may be present particularly in cases not evolving to satisfaction despite proper treatment^27^, ^28^, ^29^.

The initial conduct in the two cases of PFP from AOM was to perform a myringotomy with ventilation tube insertion (in addition to antibiotics and steroids). One of them did not evolve well (patient with WG) and was referred to closed mastoidectomy. Both improved satisfactorily from PFP as described in the results. The patients diagnosed with CCOM had grade III and grade IV PFP and underwent canal wall down mastoidectomy. The injury was located in the tympanic segment in one case and in the transition of the tympanic to the mastoid (second knee) segment in the other. Six months into follow-up the patient with grade IV PFP moved down to grade III and the other remained with grade III PFP.

PFP prognosis varies whether the patient has AOM or CCOM. When PFP is caused by AOM, proper antibiotics along with myringotomy to drain the purulent material and reduce bacteria counts lead to good nerve function recovery in most cases. On the other hand, when the cause is COM (with or without cholesteatoma), surgery is the treatment of choice. The infection around the nerve needs to be removed and function recovery is uncertain^12^, as seen in our cases.

### Labyrinthitis

There were three cases of serous labyrinthitis (all described previously) followed by other intratemporal complications, namely labyrinthine fistula, mastoiditis, and PFP. Complaints of hypacusis, tinnitus, and vertigo were present in all patients, along with moderate to severe mixed hearing loss. It was the least frequent complication seen in the study, with an annual incidence rate of 0.1%.

Sensorineural hearing loss and vestibular disorders secondary to penetration of toxins or bacteria in the inner ear structures still challenge ENTs^30^. Labyrinthitis is categorized as suppurative or serous. The first occurs due to invasion of the labyrinth by microorganisms (usually bacteria), introducing cochlear and vestibular injury and usually resulting in permanent severe or profound hearing loss, which was not seen in our study. When labyrinthine damage is caused by toxic bacteria products or inflammatory particles, the condition is called serous labyrinthitis and auditory sequelae tend to be milder and non-permanent^31^, with patients usually being able to partially recover their hearing thresholds after the infection subsides, as seen in our study.

The differentiation between serous and suppurative labyrinthitis is done clinically, based on response to treatment and audiometric improvement seen in patients with serous labyrinthitis^30^. Suppurative labyrinthitis is less prevalent today. Its incidence is estimated to have been reduced sevenfold since the introduction of antibiotics^31^.

Although serous labyrinthitis has been reported as one of the most common complications in otitis media^32^, only three subjects had it in our study. It has been assumed that many patients with serous labyrinthitis associated with

OM respond well to conventional therapy and elective ear surgery to treat the baseline disease and, consequently, diagnosis is often missed.

Insults to the labyrinth may occur in many different ways. Inflammatory processes of the meninges or the cerebrospinal fluid may travel to the inner ear (labyrinth) through the cochlear aqueduct or the internal acoustic canal (IAC). Ten percent of the patients with bacterial meningitis have labyrinthitis and profound hearing loss. Infection may also occur the other way around, i.e., it may start in the labyrinth and travel to the central nervous system. Other frequent dissemination pathways are seen when the focus of infection is in the middle ear and labyrinthine injury occurs through the round or oval window. We believe this was the mode of action of the labyrinthitis cases seen in this study. Bacteria from systemic infection may have reached the labyrinth transported by the bloodstream, in a rare hematogenic dissemination pattern^30^, ^31^.

## CONCLUSION

The incidence of intratemporal complications is significant when compared to the rates seen in developed countries.

Chronic cholesteatomatous otitis media is the most common etiology of intratemporal complications.

Labyrinthine fistula is the most common intratemporal complication. Hypacusis and tinnitus were observed in all cases.
